# Droplet and Aerosol Generation With Endonasal Surgery: Methods to Mitigate Risk During the COVID-19 Pandemic

**DOI:** 10.1177/0194599820949802

**Published:** 2020-08-11

**Authors:** Harish Dharmarajan, Monika E. Freiser, Edward Sim, Devi Sai Sri Kavya Boorgu, Timothy E. Corcoran, Eric W. Wang, Paul A. Gardner, Carl H. Snyderman

**Affiliations:** 1 Department of Otolaryngology, University of Pittsburgh Medical Center, Pittsburgh, Pennsylvania, USA; 2 University of Pittsburgh School of Medicine, University of Pittsburgh, Pittsburgh, Pennsylvania, USA; 3 Division of Pulmonary, Allergy and Critical Care Medicine, University of Pittsburgh, Pittsburgh, Pennsylvania, USA; 4 Department of Neurosurgery, University of Pittsburgh Medical Center, Pittsburgh, Pennsylvania, USA

**Keywords:** endonasal, skull base, aerosol, droplet, COVID-19, prevention, safety

## Abstract

**Objective:**

To define the aerosol and droplet risks associated with endonasal drilling and to identify mitigation strategies.

**Study Design:**

Simulation series with fluorescent 3-dimensional (3D) printed sinonasal models and deidentified cadaveric heads.

**Settings:**

Dedicated surgical laboratory.

**Subjects and Methods:**

Cadaveric specimens irrigated with fluorescent tracer and fluorescent 3D-printed models were drilled. A cascade impactor was used to collect aerosols and small droplets of various aerodynamic diameters under 15 µm. Large droplet generation was measured by evaluating the field for fluorescent debris. Aerosol plumes through the nares were generated via nebulizer, and mitigation measures, including suction and SPIWay devices, nasal sheaths, were evaluated regarding reduction of aerosol escape from the nose.

**Results:**

The drilling of cadaveric specimens without flexible suction generated aerosols ≤3.30 µm, and drilling of 3D sinonasal models consistently produced aerosols ≤14.1 µm. Mitigation with SPIWay or diameter-restricted SPIWay produced same results. There was minimal field contamination in the cadaveric models, 0% to 2.77% field tarp area, regardless of drill burr type or drilling location; cutting burr drilling without suction in the 3D model yielded the worst contamination field (36.1%), followed by coarse diamond drilling without suction (19.4%). The simple placement of a flexible suction instrument in the nasal cavity or nasopharynx led to complete elimination of all aerosols ≤14.1 µm, as evaluated by a cascade impactor positioned immediately at the nares.

**Conclusion:**

Given the findings regarding aerosol risk reduction, we strongly recommend that physicians use a suction instrument in the nasal cavity or nasopharynx during endonasal surgery in the COVID-19 era.

The risks associated with endonasal surgery and transmission of severe acute respiratory syndrome coronavirus 2 (SARS-CoV-2) remain unclear. Several modes of SARS-CoV-2 transmission have been proposed, including direct contact, droplets, and aerosols.^1,2^ In general, aerosols with an aerodynamic diameter <5 µm can reach the alveoli, and particles <10 µm can penetrate below the glottis. In addition, particles between 10 and 20 µm settle more readily, and particles >20 µm have a ballistic trajectory.^3^ In this study, aerosols are defined as particles with an aerodynamic diameter of ≤10 µm, whereas droplets are defined with diameters >10 µm. This is in accord with differences in fluid dynamics between the 2 groups regarding suspension time and deposition in different airway regions.^3^ Given that endoscopic drilling is a fundamental tool in rhinology and skull base surgery, it is important to understand any associated aerosolization risks. Recently, Workman et al^4^ identified endonasal drilling as the greatest risk of aerosol generation using an optical particle sizer. However, there are limited data in the literature regarding aerosolization risk with otolaryngology procedures and interventions that mitigate aerosol risk. Our project aims to define the aerosol and droplet risks associated with endonasal drilling using a cascade impactor. This study evaluates the aerosol dispersion from endonasal drilling and explores potential mitigation measures for aerosol and droplet spread.

## Materials and Methods

Overall, there are 3 main components of the study: (1) field contamination survey assessing the distribution of fluorescent contamination on the surgical field and on the provider’s personal protective equipment (study of large visible droplets), (2) simulation of sinonasal aerosol dynamics with nebulized vitamin B2 solution and gross visualization of mitigation measures, and (3) cascade impactor studies to specifically record the presence of small droplet and aerosol particles generated under various simulation scenarios and after application of mitigation measures (see Suppl. Video S1 in the online version of the article). The field contamination survey addresses large droplet risk while the cascade impactor trials assess aerosol and small droplet risk (<15 µm) associated with endonasal drilling. The simulations of aerosol dynamics highlight how various mitigation measures, including suction use and nasal aperture reduction, can affect aerosol escape through the nares.

### Reagents and Specimens

This study was conducted with approval from the University of Pittsburgh Committee for Oversight of Research and Clinical Training Involving Decedents (CORID #888). Vitamin B2 (riboflavin) was used as the fluorescent tracer for all simulation trials. Both 0.05-g/L and 1-g/L (in 0.9% normal saline) vitamin B2 solutions were prepared for use as drilling irrigation as well as for aerosol generation via an AeroEclipse II Breath Actuated Nebulizer (BAN) (Trudell Medical International).^5^ The 1-g/L concentrated solution was used in all trials except for the field contamination trials, in which the 0.05-g/L solution was used. The BAN system was used to create vitamin B2 aerosol particles with an average mass median aerodynamic diameter (MMAD) of 2.8 µm.^6,7^ A combination of fluorescent 3-dimensional (3D) printed sinonasal models created from Formlabs white resin (Formlabs) and deidentified cadaveric heads was used for the endonasal surgery simulations. We chose to use models and specimens in which there was a partial septectomy defect in order to best simulate skull base surgery, in which endonasal drilling is most often performed in the setting of a wide surgical cavity. SPIWay nasal sheaths (SPIWay, LLC)^8^ were tested as potential mitigation methods. The SPIWay is an endonasal sheath used to reduce mucosal trauma during endoscopic procedures and to avoid contamination of scope lenses by surrounding debris.

A Next Generation Impactor (NGI; Copley Scientific) was used to collect aerosol and small droplet particles based on specific aerodynamic sizes as they exited from the nares.^9,10^ This was used to determine the aerodynamic size distribution present with each simulation scenario.

### Experimental Setup

#### Field contamination study

**Figure 1.**
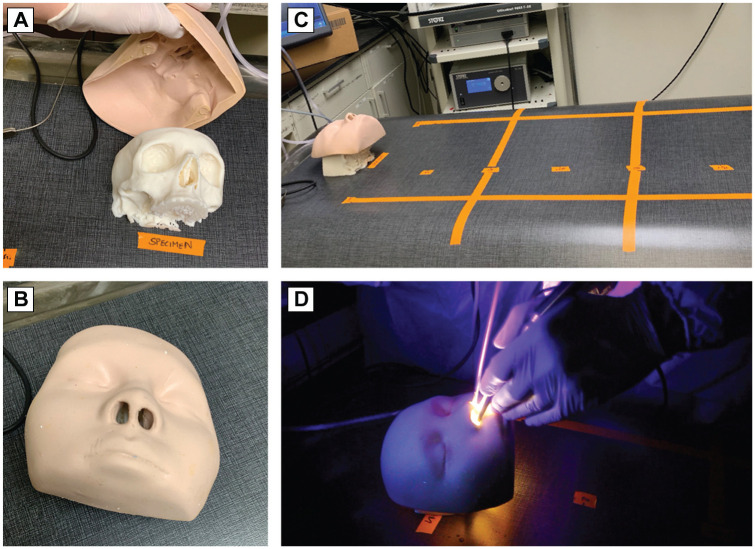
Field contamination setup. (A, B) Fluorescent 3-dimensional (3D) model with and without face covering. (C) The 3D model or cadaveric specimen was placed on top of a tarp with 6-inch distances marked. After each 2-minute trial, the tarp was examined for debris. (D) 3D model demonstrating drilling conditions.

The specimens, 3D models with an overlying mask or cadaver heads, were placed at the edge of a black tarp, which was labeled with 6-inch increment markers using orange tape (**Figure 1**). Two providers participated in each trial, with one handling the endoscope and the other using the drill. Prior to each trial, the tarp was cleaned and providers’ personal protective equipment (PPE) was changed; both were checked to ensure no baseline fluorescence. Five total trials were performed to test whether the following variables affected the degree and pattern of field contamination on the tarp and providers’ PPE: drill burr type (6-mm cutting burr vs 4-mm coarse diamond burr), use of vitamin B2 irrigation (0.05 g/L solution), drilling site (clivus, Draf III, sphenoid), and use of rigid suction (9 Fr). Each scenario was simulated once for a total duration of 2 minutes.

#### Simulation of sinonasal aerosol dynamics

For these trials, a cadaver head was intubated retrograde with an 8.0 endotracheal tube (ETT) with the distal tip projecting into the nasopharynx as confirmed with an endoscope. Aerosols were generated using a BAN nebulizer with a 1-g/L vitamin B2 solution. Under room light conditions, the aerosol plumes could be visualized exiting the nares; this was accentuated under UV blacklight. Different variables were studied to evaluate whether the aerosol plumes could be reduced or eliminated altogether: presence of suction, location of suction tip (inside vs outside nasal cavity), type of suction (rigid vs flexible), finger occlusion on rigid suction channel, use of SPIWay nasal sheath,^8^ and simulated nasal aperture size reduction using a combination of orthodontic rubber bands wrapped around SPIWays to narrow the SPIWay lumen and placement of multiple endoscopic instruments within the SPIWay lumen to obstruct the nasal cavity. The orthodontic elastics were wrapped twice around the SPIWay at the nares to greatly reduce the diameter of the instrument aperture with the concept of reducing aerosol escape through the SPIWay but allowing enough space for instrument passage.

#### Impactor study

The Next Generation Impactor (NGI) was used to determine the presence of fine droplets and particles for each simulation scenario.^10^ The NGI impactor has 8 sequential stages for collecting aerosols of different aerodynamic diameter sizes based on inertial impaction. Aerodynamic diameter relates to the settling properties of the aerosol and indicates that the aerosol has equivalent properties to a water density aerosol of that size. It is clinically relevant in that it reflects where a particle is most likely to collect in the airway. Smaller particles will have a lower tendency to be collected by impaction while larger particles will be impacted against a solid surface (filter stage).^11^ Aerosol deposition is influenced by multiple aerosol characteristics, including density and shape.^12^ The D_50_ refers to the cutoff diameter where collection efficiency of the cascade impactor is 50%.^12^ Unlike optical particle sizers, impactors provide a direct measurement of aerodynamic particle size^11^ based on the momentum of individual particles (product of density and velocity).

**Figure 2.**
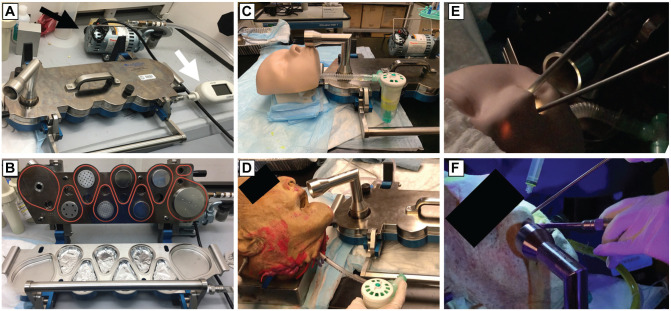
Impactor survey setup. (A) Impactor setup with vacuum generator (black arrow) and flow meter (white arrow). (B) Opened view of Next Generation Impactor (NGI) with aluminum foils in collection chambers. Experimental setup demonstrating nebulizer conditions with 3-dimensional (3D) model (C) and cadaver head (D) with the impactor inlet just inferior and anterior to the nostrils. Experimental setup demonstrating drilling conditions with 3D model (E) and cadaver head (F).

**Figure 3.**
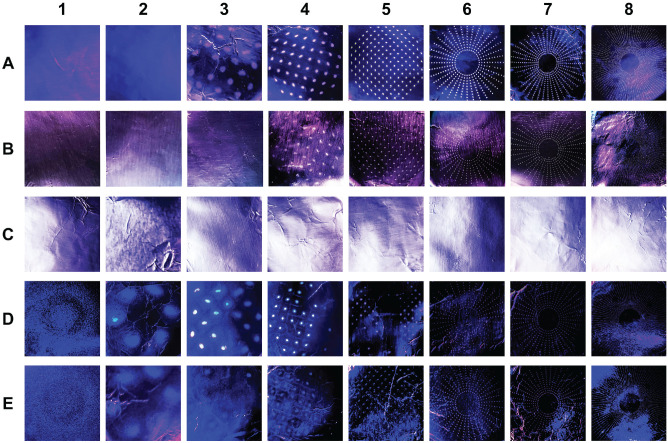
Representative images of filter foil results from impactor trials. Photographs of removable foil pieces that lined the cascade impactor capture chambers illuminated under UV light for nebulized vitamin B2 trial (positive control; A), cadaver coarse diamond drilling with vitamin B2 irrigation without suction (B), cadaver coarse diamond drilling with vitamin B2 irrigation and suction use (C), 3-dimensional (3D) model cutting burr drilling with nebulized vitamin B2 without suction (D), and 3D model coarse diamond burr drilling with nebulized vitamin B2 without suction (E). Particles filtered based on average aerodynamic diameter into 8 impactor stages are displayed: (1) 14.1 µm, (2) 8.61 µm, (3) 5.39 µm, (4) 3.30 µm, (5) 2.08 µm, (6) 1.36 µm, (7) 0.98 µm, and (8) <0.98 µm.

The NGI was connected to a flow meter and a vacuum source generating a 15-L/min inlet flow rate, which was used for all trials. The NGI inlet nozzle was positioned as close as possible to specimen’s nares to create a closed system and minimize losses in detecting the generated particles (**Figure 2**). Each stage of the NGI is calibrated to a specific aerodynamic particle diameter (D_50_, µm) based on the flow rate: stage 1, 14.1; stage 2, 8.61; stage 3, 5.39; stage 4, 3.30; stage 5, 2.08; stage 6, 1.36; and stage 7, 0.98. Stage 8 is the micro-orifice collector (MOC) filter—smallest particles without a defined D_50_ value.^13^ Each filter stage has a corresponding capture chamber. Here, these were lined with aluminum foil to prevent accumulation of fluorescent material between experiments. UV light exposed the presence of any filtered particulate matter on the foil pieces. Between each trial, the impactor inlet nozzle, filter tray, and individual chambers were cleaned with distilled water and new aluminum foils were placed into collection chambers. Nebulized vitamin B2 solution (1 g/L) was used as a positive control to test the NGI (**Figure 3**). For the negative control, the NGI was left to run sampling room air; this did not pick up any detectable aerosols in the collection chambers. Each impactor trial was standardized to 2 minutes and performed once for each scenario, combination of variables, being studied. The impactor trials focused on a variety of scenarios using 3D models as well as cadaveric heads to assess baseline aerosol risk and efficacy of mitigation measures. When an impactor trial involved drilling, the clivus was used as the drilling site. For baseline risk assessments, a 1-g/L vitamin B2 irrigation solution was used in the setting of drilling; nebulized vitamin B2 was used to study mitigation measures as this produced an exaggerated or worst-case scenario for aerosol generation. The combination of trials evaluated the impact of the following variables on aerosol risk: burr type (cutting vs coarse), presence and location of suction tip, and reduction of nasal aperture size (SPIWay with 3/16-inch orthodontic elastics and instruments).

## Results

### Field Contamination Survey

**Figure 4.**
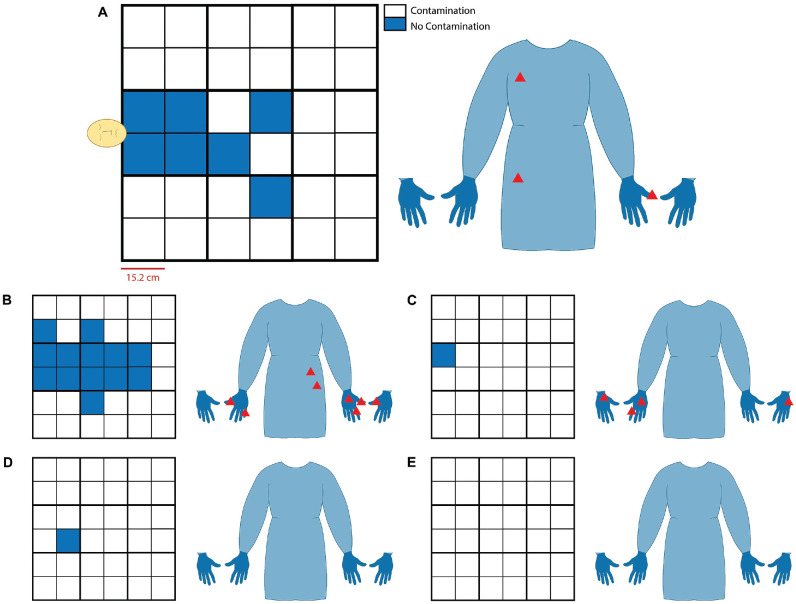
Field contamination results. Representative grids and personal protective equipment (PPE) displaying field contamination present on tarp and provider PPE (gown and palmar and dorsal surfaces of gloves), respectively, following each trial. Each grid square represents 6-inch units with the model head oriented on the center of the left border of the grid (A). This scale and orientation were kept consistent across all images. Coarse diamond burr without suction (A) and cutting burr without suction (B) on fluorescent 3-dimensional model. Cutting burr drilling Draf III (C), cutting burr drilling clivus (D), and coarse diamond burr drilling Draf III (E) on cadaver head. All cadaver trials were with suction at the discretion of the surgeon.

The first trial performed was on the fluorescent 3D model using a 6-mm cutting burr with no suction or irrigation. In this scenario, there were visible particles and smoke emanating from the nose during drilling. At the end of the trial, 36.1% of the tarp was contaminated, with most particles noted within the first 2.5 feet of the model (**Figure 4B**). The 4-mm coarse diamond burr was used next with no suction or irrigation. The amount of debris was visibly less as compared to the 6-mm cutting burr, and observed particulate matter was smaller in size. The contamination covered 19.4% of the tarp, and the distribution was similar to that of the 6-mm cutting burr at around 2 feet (**Figure 4A**). There was a small amount of particulate matter on the gloves and gown of the drilling surgeon for both trials (**Figure 4A**,**B**).

Next, cadaver trials were performed. As compared to trials using 3D models, there was minimal field contamination with the cadaveric trials. The nasal cavity was irrigated with B2 solution prior to drilling and then intermittently reapplied during drilling. Draf III drilling with the cutting burr had minimal debris noted on the tarp (**Figure 4C**), as did clival drilling with the cutting burr (**Figure 4D**), with both trials contaminating 2.77% of the field. Draf III drilling with the coarse diamond burr produced intermittent smoke with no visible tarp or surgeon contamination (**Figure 4E**). Rigid suction was used during each trial. Smoke was not noted when the suction was used.

### Sinonasal Aerosol Fluid Dynamics Simulation

Nebulized vitamin B2 produced significant aerosol plumes emanating from the nares (see Suppl. Video S1 in the online version of the article). Aerosol plumes disappeared when a flexible suction (14 Fr) was placed inside the nasal cavity at either shallow or deep depths or parked in the nasopharynx. Placement of the flexible suction at the columella was effective as most of the aerosol plumes were still drawn to the suction opening; however, plume escape was noted if the suction tip was moved just off midline favoring 1 naris. Similarly, with use of a rigid suction (9 Fr) and thumb on the relief hole, the aerosol plumes disappeared whenever the tip was inside the nasal cavity or nasopharynx or even directly at the columella. Without occluding the relief hole, the rigid suction still eliminated all visible aerosol plumes when placed inside the nasal cavity or nasopharynx but failed when suction tip was placed just outside the nares.

Directing nebulized vitamin B2 plumes into a SPIWay did not show any leak through the material of the SPIWay itself. When SPIWays were positioned in the nasal cavity, there were visible aerosol plumes still coming out of the nares but in a focused, nondispersed manner due to the SPIWay outer flange. When orthodontic elastics were applied to narrow the SPIWay lumen, plume volume decreased but was not eliminated. This remained the case when instruments were introduced after constricting the SPIWay opening. Placement of a rigid endoscope and a nonfunctioning rigid suction in the left naris and a drill in the right naris almost completely blocked the banded SPIWays and produced a significant decrease in aerosol plume escape (see Suppl. Video S1 in the online version of the article). However, with any manipulation of the instruments in the lateral or vertical axis, the plumes would escape around the SPIWay itself. No combination of rubber band application was found to completely prevent aerosol plume escape, nor was pushing the SPIWay outer flanges immediately inside the nares. When a flexible suction was parked underneath a nonconstricted SPIWay in the nasopharynx, the aerosol plumes disappeared. The SPIWay device effectively secured the flexible suction tip in the posterior nasal cavity without compromising full expansion of the SPIWay device.

### Impactor Studies for Baseline Risk and Mitigation Measures

Nebulized vitamin B2 was applied to the fluorescent blue 3D models (via retrograde intubation into nasopharynx), and the models were drilled with coarse diamond and cutting burrs during nebulization. With both drill bits, filters 1 to 8 were positive (**Table 1**). The particles in filters 1 to 4 were fluorescent blue, indicating bone dust, whereas the particles in filters 5 to 8 were fluorescent yellow, indicating vitamin B2 presence. When using the cutting burr as opposed to coarse diamond burr, there was noticeably more fluorescent blue bone dust in filters 1 to 4. Alternatively, when using the coarse diamond burr, there were greater fluorescent yellow particles in filters 5 to 8, as compared to trials with the cutting burr. The cutting burr generated particles with a larger mass median aerodynamic diameter compared to the coarse diamond burr. When a flexible suction was parked inside the naris, both bone dust and the vitamin B2 aerosols were eliminated, with no particles detected in filters 1 to 8.

**Table 1.** Three-Dimensional Printed Sinonasal Model Trials.

Trial			Particle Size (µm): D_50_ at 15 L/min								
Drill	Suction	B2 Tracer	14.1	8.61	5.39	3.30	2.08	1.36	0.98	<0.98
NA	NA	Nebulized B2	–	–	**+**	**+**	**+**	**+**	**+**	**+**
NA	Flexible suction^a^	Nebulized B2	—	—	—	—	—	—	—	—
NA	NA	Nebulized Saline	—	—	—	**+**	**+**	**+**	**+**	**+**
Coarse burr	NA	Nebulized B2	*	*	*	*	**+**	**+**	**+**	**+**
Cutting burr	NA	Nebulized B2	**	**	**	*/+	**+**	**+**	**+**	**+**
Cutting burr	Flexible suction^a^	Nebulized B2	—	—	—	—	—	—	—	—

Abbreviations: NA, not applicable; *, presence of blue fluorescent bone dust; **, increased presence of blue fluorescent bone dust; +, presence of particle aggregates; –, no particles present at the specific impactor stage.

a Flexible suction was parked approximately 6 cm away from nasal aperture.

To assess maximum baseline aerosol risk, a cadaver head was drilled using a coarse diamond drill with intermittent vitamin B2 irrigation but without a parked suction. In this scenario, the impactor detected aerosol particles in filters 4 to 8 (**Table 2**). It was difficult to distinguish which filters had cadaveric bone dust as it was not fluorescent and did not have a tracer. With the addition of SPIWay devices, aerosol particles were still noted in filters 4 to 8 (**Table 3**). This remained the case when the SPIWays were constricted by rubber bands. Application of a flexible suction inside the naris eliminated all aerosol particles detected by the NGI with negative filters 1 to 8.

**Table 2.** Cadaver Trials and Suction Mitigation.

Trial			Particle Size (µm): D_50_ at 15 L/min							
Drill	Suction	B2 Tracer	14.1	8.61	5.39	3.30	2.08	1.36	0.98	<0.98
NA	NA	Nebulized B2	—	—	**+**	**+**	**+**	**+**	**+**	**+**
NA	Flexible suction in nasopharynx	Nebulized B2	—	—	—	—	—	—	—	—
NA	Flexible suction at columella	Nebulized B2	—	—	—	—	—	—	—	—
Coarse diamond	NA	B2 irrigation	—	—	—	**+**	**+**	**+**	**+**	**+**
Coarse diamond	Flexible suction	B2 irrigation	—	—	—	—	—	—	—	—

Abbreviations: NA, not applicable; +, presence of particle aggregates; –, no particles present at the specific impactor stage.

**Table 3.** Cadaver Trials and Mitigation With Nasal Aperture Reduction.

Trial				Particle Size (µm): D_50_ at 15 L/min							
Drill	SPIWay	Suction	B2 Tracer	14.1	8.61	5.39	3.30	2.08	1.36	0.98	<0.98
Coarse diamond	NA	NA	B2 irrigation	—	—	—	+	+	+	+	+
Coarse diamond	Bilateral	NA	B2 irrigation	—	—	—	+	+	+	+	+
Coarse diamond	Bilateral with rubber band	NA	B2 irrigation	—	—	—	+	+	+	+	+
Coarse diamond	Bilateral	Flexible suction	B2 irrigation	—	—	—	—	—	—	—	—
NA	NA	NA	Nebulized B2	—	—	+	+	+	+	+	+
NA	Bilateral	NA	Nebulized B2	—	—	+	+	+	+	+	+
Drill on right (inactive); endoscope on left	Bilateral with bunny rubber bands	Inactive rigid suction on left	Nebulized B2	—	—	+	+	+	+	+	+
NA	Bilateral	Flexible suction	Nebulized B2	—	—	—	—	—	—	—	—

Abbreviations: NA, not applicable; +, presence of particle aggregates; –, no particles present at the specific impactor stage.

Mitigation measures using nebulized vitamin B2 with cadaveric specimens were also assessed (**Tables 2** and **3**). As a positive control, nebulized vitamin B2 aerosol plumes were detected by the impactor as fluorescent particles in filters 3 to 8. Addition of bilateral SPIWays resulted in filters 3 to 8 still positive for aerosols. When the SPIWays were constricted with double-wrapped 3/16-inch 3.5-oz orthodontic elastics and instruments were placed in addition (endoscope and inactive rigid suction in left naris and drill in right naris), there were still aerosol particles detected in filters 3 to 8. However, when a flexible suction was placed inside the naris at the 6-cm mark in addition to the existing SPIWays, there were no aerosols detected in filters 1 to 8.

## Discussion

The potential for aerosol generation with endonasal surgery, especially with power instrumentation, is not well defined in the literature. Several experimental methods are available for measuring the aerosols and droplets generated with endonasal procedures. For this project, a cascade impactor was used to directly separate particles based on the aerodynamic diameter, a function of individual particle density and shape.^13^ This method evaluated the production and mitigation of aerosols of 8 different sizes under 14.1 microns. A recent study also investigated aerosol generation from endonasal surgery using an optical particle sizer, which was calibrated to detect particles between 0.3 and 10 microns using laser diffraction analysis but did not separate or collect aerosols by aerodynamic diameter.^4^ The optical particle sizer is an efficient tool to detect aerosols. However, it is an indirect method that requires approximating the refractive index of a heterogeneous mix of aerosol particles; it is not possible to evaluate the aerodynamic diameter with the optical particle sizer.

This study aimed to accurately define aerosol risk with endonasal drilling based on aerodynamic diameter and explore mitigation strategies. In the cadaver specimens, aerosols with D_50_≤3.30 µm were generated by drilling with a coarse diamond burr and limited irrigation. With the fluorescent 3D models, the coarse diamond burr resulted in greater distribution of fine aerosol particles (filters 5-8; ≤2.08 µm), whereas the cutting burr produced a greater distribution of larger aerosols (filters 1-4; 3.30-14.1 µm). Reduction of the cadaveric anterior nasal aperture using banded SPIWay sheaths still yielded detectable aerosols under 3.30 µm. However, use of a flexible suction parked inside the naris, outside the SPIWay device, resulted in elimination of all detectable aerosols under 14.1 µm (filters 1-8).

This study proves that placing a suction inside the nasal cavity or nasopharynx significantly reduces aerosols. This was demonstrated both visually with sinonasal fluid dynamics simulations and objectively with the cascade impactor trials. All trials in which a suction was parked inside of the nose demonstrated only negative results. This was consistent with every condition that was aerosol generating, including the cadaver endonasal drilling trials, the 3D model drilling trials, and the nebulized vitamin B2 fluid dynamics simulations. The nebulized vitamin B2 trials provided a “worst-case scenario” in which aerosols were constantly produced, and even in these conditions, the suction resulted in a completely negative result with all 8 NGI filters empty.

Regardless of suction type, rigid or flexible, or depth of suction tip placement in the nasal cavity or nasopharynx, aerosol particles across a range of aerodynamic diameters were successfully removed from the surgical field and were prevented from exiting the nares. If the suction tip was placed outside of the nasal cavity centered at the columella, this still resulted in aerosol mitigation; however, even slight mispositioning of the suction tip off to one side caused aerosol escape from the contralateral nostril. Based on the findings of this study, we strongly recommend that physicians performing endonasal surgery place a suction instrument in the nasal cavity or nasopharynx to mitigate aerosol risk. Provided that the suction tip is maintained open during the case and not occluded by tissue, the aerosol plumes will be directed toward the suction tip rather than exiting the nares. Reducing aerosol production may lead to safer conditions for surgeons and operating room staff when treating a patient with active SARS-CoV-2 infection. Our current practice has been adapted to include a flexible tracheal suction catheter trapped between the SPIWay device and nasal cavity, therefore held outside the path of instrument passage, in addition to a standard 2-surgeon, 4-hands technique with rigid suction at all times during drilling.

In terms of droplet risk, the amount of debris noted in the field after drilling varied depending on whether a coarse diamond or cutting burr was used. The 3D model trials provided a fluorescent tracer to easily visualize particles on the field and in the air (**Figure 4**). A cutting burr produced larger particles and more debris. A coarse diamond burr produced finer particles, less debris in total, and more visible smoke during drilling. With the cadaver field contamination studies, there was minimal to no debris on the field after using the cutting burr to drill Draf III or clivus and using diamond burr to drill Draf III. We attribute this to rigid suction use during the cadaveric trials, which was performed to most closely simulate operating room conditions, and the varying characteristics of 3D model resin vs human bone.

Several groups have previously studied the aerosol and droplet risks associated with endonasal surgery^4,14^ as well as mitigation procedures, including use of an isolation drape,^15^ negative airway pressure respirator,^16^ and concurrent suction with procedures.^4,17^ Our results demonstrate that there is a baseline aerosol risk associated with endonasal drilling as previously shown by Workman et al^4^; however, in contrast to their results, we found that suction use eliminates aerosols altogether regardless of suction position in the nasal cavity or nasopharynx. The difference in results may be attributed to the experimental setup. In these trials, the method of aerosol detection is extremely important given the small diameter and quantity of particles. The ideal aerosol detection system will have the following principles: (1) direct evaluation of a particle’s aerodynamic diameter using physical measurements of size and momentum; (2) consideration of each particle’s shape, size, and density, which all influence aerodynamic diameter; (3) minimization of detection error (aerosol loss to surroundings in a closed system or lack of proper sampling when using indirect measures in an open setting); (4) reliable measurement of particle concentration; and (5) ability to link aerosols to the specimen via a specific tracer.

Our experimental method presents several advantages. The cascade impactor allowed for direct determination of aerosol aerodynamic size, which has not been addressed by any study in the otolaryngology literature. Even though individual particle size, shape, and density were unknown, the impactor’s inertial filtering system enabled us to determine approximate aerodynamic diameters based on known device calibrations at 15.0 L/min. We attempted to mimic a closed system by ensuring that the impactor nozzle was almost flush against the nares; this positioning allowed for maximizing detection of all generated aerosols and limiting aerosol escape into the surroundings. A high flow rate (15.0 L/min) ensured that even aerosols suspended inside of the nasal cavity or nasopharynx were likely to be detected by the impactor. Unlike other studies, we were able to collect the aerosol particles based on aerodynamic size so that it could be used for biochemical analysis if needed. The addition of vitamin B2 as a fluorescent tracer allowed us to link the aerosols to the original specimen rather than to the provider or the surrounding environment. Our study presents the novel use of vitamin B2 as a fluorescent tracer in aerosol contamination studies and the utility of an impactor in aerosol contamination models with an emphasis on filtering aerosol particles produced in otolaryngology procedures.

There are a few limitations to our study. Although it was easy to distinguish the presence or absence of aerosols with vitamin B2 fluorescence, we did not use a spectrophotometer to precisely quantify the relative fluorescence of each impactor stage, which showed visible collection of particles. This study does not address whether the captured aerosols were biologically active with the capacity to cause infection. We are working on further trials to determine whether aerosol particles isolated from procedures in SARS-CoV-2–positive patients have infectious potential. Despite the unclear baseline risk, we urge physicians performing endonasal surgery to use endonasal suction placement as it is a ubiquitous and effective tool to mitigate aerosol risk.

Our current practice for skull base cases has been adapted to place a flexible tracheal suction catheter in the nasopharynx; the suction tubing is situated between the SPIWay sleeve and nasal cavity surface, and therefore held outside the path of instrument passage. In addition, a handheld suction is used during each case so between the 2 suctions, there is always an active suction in the nasopharynx. For endoscopic sinus surgery cases when the SPIWay sleeve is not routinely used, the flexible suction tip is parked just inside of the nostril and held in place with tape. Some surgeons at our institution choose to park the suction in the nasopharynx or deeper in the nasal cavity for endoscopic sinus surgery cases. Using a parked suction does require having an extra suction set up but has not presented any difficulties when operating, including in patients with a bleeding tendency or diseased sinuses. The flexible suction can be maintained for the duration of each case without problems. The addition of a parked flexible suction to the operative setup is quick, inexpensive, and reliable. We hope this becomes the accepted standard for cases involving endonasal instrumentation, especially with drilling.

## Conclusion

This study evaluates aerosol production during endonasal drilling based on aerodynamic diameter and explores mitigation strategies. Across a range of drilling scenarios, aerosols under 15 µm were consistently generated. The simple placement of a suction instrument in the nasal cavity or nasopharynx led to complete elimination of all detectable aerosols, as evaluated by a cascade impactor positioned immediately at the nares. Given the findings in our study, we strongly recommend that physicians use a suction instrument in the nasal cavity or nasopharynx during endonasal surgery to mitigate aerosol risk.
